# Antiproliferative and Apoptotic Effects of Graphene Oxide @AlFu MOF Based Saponin Natural Product on OSCC Line

**DOI:** 10.3390/ph15091137

**Published:** 2022-09-12

**Authors:** Seyyed Mojtaba Mousavi, Seyyed Alireza Hashemi, Yasmin Ghahramani, Rouhollah Azhdari, Khadijeh Yousefi, Ahmad Gholami, Fatemeh Fallahi Nezhad, Neralla Vijayakameswara Rao, Navid Omidifar, Wei-Hung Chiang

**Affiliations:** 1 Department of Chemical Engineering, National Taiwan University of Science and Technology, Taipei City 106335, Taiwan; 2 Nanomaterials and Polymer Nanocomposites Laboratory, School of Engineering, University of British Columbia, Kelowna, BC V1V 1V7, Canada; 3 Department of Endodontics, Shiraz University of Medical Sciences, Shiraz 71956-15787, Iran; 4 Biotechnology Research Center, Shiraz University of Medical Sciences, Shiraz 71468-64685, Iran; 5 Department of Dental Materials and Biomaterials Research Centre, Shiraz Dental School, Shiraz University of Medical Sciences, Shiraz 71956-15787, Iran; 6 Department of Pathology, Shiraz University of Medical Sciences, Shiraz 71468-64685, Iran

**Keywords:** aluminum fumarate, saponin, squamous cell carcinoma cancer, graphene oxide

## Abstract

The increasing rate of oral squamous cell carcinoma (OSCC) and the undesirable side effects of anticancer agents have enhanced the demand for the development of efficient, detectable, and targeted anticancer systems. Saponins are a diverse family of natural glycosides that have recently been evaluated as an effective compound for the targeted therapy of squamous cell carcinoma. Due to their porous nature and stable structure, metal–organic frameworks (MOFs) are a well-known substance form for various biological applications, such as drug delivery. In this study, we fabricated a novel hybrid, highly porous and low-toxic saponin-loaded nanostructure by modifying graphene oxide (GO)/reduced GO (rGO) with aluminum fumarate (AlFu) as MOF core–shell nanocomposite. The characterization of the nanostructures was investigated by FTIR, TEM, EDX, FESEM, and BET. MTT assay was used to investigate the anticancer activity of these compounds on OSCC and PDL normal dental cells. The effect of the nanocomposites on OSCC was then investigated by studying apoptosis and necrosis using flow cytometry. The GO/rGO was decorated with a saponin–AlFu mixture to further investigate cytotoxicity. The results of the MTT assay showed that PDL cells treated with AlFu–GO–saponin at a concentration of 250 μg/mL had a viability of 74.46 ± 16.02%, while OSCC cells treated with this sample at a similar concentration had a viability of only 38.35 ± 19.9%. The anticancer effect of this nanostructure on OSCC was clearly demonstrated. Moreover, the number of apoptotic cells in the AlFu–GO–saponin and AlFu–rGO–saponin groups was 10.98 ± 2.36%–26.90 ± 3.24% and 15.9 ± 4.08%–29.88 ± 0.41%, respectively, compared with 2.52 ± 0.78%–1.31 ± 0.62% in the untreated group. This significant increase in apoptotic effect observed with AlFu–rGO–saponin was also reflected in the significant anticancer effect of saponin-loaded nanostructures. Therefore, this study suggests that an effective saponin delivery system protocol for the precise design and fabrication of anticancer nanostructures for OSCC therapy should be performed prior to in vivo evaluations.

## 1. Introduction

Oral squamous cell carcinoma (OSCC) is the most common oral neoplasm, accounting for more than 90% of all oral malignancies and 38% of head and neck cancers. Despite improved treatment strategies, morbidity and mortality rates for OSCC have not changed significantly over the past 30 years. The mortality and morbidity rates are 6.6/100,000 and 3.1/100,000, respectively, for males and 2.9/100,000 and 1.4/100,000, respectively, for women. OSCC is also increasingly common in young white people aged 18 to 44 years, especially white women. Patients with OSCC typically have a 5-year survival rate of 40% to 50%. The 2021 National Comprehensive Cancer System guidelines for non-nasopharyngeal malignancies list the following procedures for treating OSCC after surgery. Cisplatin, carboplatin, doxorubicin, and 5-fluorouracil account for the majority of systemic treatment. A nanoplatform can serve as a drug delivery system (DDS) and an imaging agent that can be detected in the body by fluorescence emission [1,2,3,4]. A DDS should overcome drug resistance, provide improved local drug delivery systems that concentrate the drug at the tumor site, such as in poorly vascularized regions, and minimize the risk of damage to adjacent normal or healthy tissue. Specifically, nanomaterials have a large surface area and small size that enable them to penetrate human cell membranes and enhance the biodistribution of anticancer drugs in targeted areas. In addition, the microenvironment, including extracellular matrix, fibrosis, and pH, can alter the properties of nanoparticles and influence their interaction with the cell membrane and ultimately their intracellular fate. For NPs targeting tumor cells, the tumor microenvironment (TME) can significantly affect cell fate [5,6,7]. Saponins are a class of amphipathic glycosides found in various plant species [8,9]. The potent effect of saponins on cancer cells is of great interest to the pharmaceutical industry and drug delivery [10,11]. By altering cell cycle proteins such as cyclins, cyclin-dependent kinases, and checkpoint proteins, these compounds have shown promising anticancer potential that can stop the spread of cancer cells [11,12,13]. Saponins are a diverse family of natural surfactants found in many plants. In addition, saponin molecules consist of a hydrophobic region called the aglycone, which is linked to one or more oligosaccharide chains (sugars) that form the hydrophilic part of the molecule. Most saponins have two sugar chains (bidesmosidic saponins), a few have one sugar chain (monodesmosidic saponin), and in rare cases, there are three sugar chains. The combination of a hydrophobic aglycone backbone and hydrophilic sugar molecules makes the saponins highly amphipathic and gives them foaming and emulsifying properties in aqueous solutions [14]. Microcapsules of saponins can assume many shapes and structures, depending on plant origin, temperature, pH, and electrolyte concentration [13,14,15]. According to their aglycone counterparts, saponins are divided into triterpenoid saponins and steroidal saponins [16,17,18]. The difference between the two groups is that the triterpenoid saponins contain 30 C atoms, while the steroidal saponins have 27 C atoms [19,20,21]. The saponin from Quillaja bark was the type of saponin used in this study. Quillaja bark saponin is mainly composed of a triterpenoid saponin with anticancer properties known as quillic acid-type aglycone [20,21,22]. Metal–organic frameworks (MOFs) are used in various fields, including gas storage and separation, molecular sieves, sensing, and catalysis. Due to their outstanding properties, MOFs are a well-known substance form for various biological applications, such as drug delivery and biosensors. They are porous materials with a high degree of organization, consisting of metallic coordination centers connected by organic linkers. Aluminum fumarate and zeolitic imidazolate frameworks (AlFu and ZIFs) have recently attracted much attention due to their exceptional surface area and porosity. These compounds typically contain imidazolate derivatives and tetrahedrally coordinated metal ions (M = Zn^2^+, Co^2^+, Cd^2^+, Mg^2^+) [23,24,25,26,27,28,29,30,31,32]. The porous MOFs often exhibit high physiological stability and considerable potential for in situ and post-synthetic functionalization with specific biomolecules, either on metals or organic ligands [33,34,35]. Immobilization of enzyme–MOF composites on permeable flexible membranes is an effective way to produce bioactive substrates. MOFs are excellent hosts for adsorption and interaction of short peptides, antibodies, and nucleic acids. MOF films are, therefore, ideal for maintaining cell activity both in vitro and in nature. In addition, MOFs can be used to fabricate a variety of structures with highly tunable pore size (typically 0.4–6 nm) and surface area (500–4500 m^2^/g) through the use of different metal groups and numerous organic compounds. Controlling the penetration of the framework can lead to different MOF configurations, porosities, and functionalities [36,37,38,39,40,41,42]. By changing the efficient parameters of the synthesis processes or by applying other methods, the size of the particles can also be determined. They have a fixed pore space that can accommodate guest molecules by changing the cell volume or the arrangement of the subnetworks. AlFu, for example, has a modified porosity character under the right pressure, or can adsorb larger guest molecules via linker rotation [43,44,45,46,47,48,49,50]. MOFs are an excellent material for simulating the mechanical and physical properties of real tissue because they have fascinating chemical and structural properties [44,45,46,47,48,49,50,51,52,53,54,55,56,57]. AlFu, graphene oxide (GO), and reduced graphene oxide (rGO) have emerged as excellent nanomaterials for the development of hybrid and multifunctional materials due to their potential. GO is characterized in this field by high drug loading, enhanced permeability and retention (EPR), pH sensitivity, and large specific surface area. Therefore, they can be integrated into various delivery systems for cancer therapies. The EPR effect leads to an increase in permeability. As a result, the nanocarriers can penetrate capillaries or leukocytes due to their tiny size (20–200 nm) and eventually concentrate in the intercellular space of the tumor. According to some studies, GO can be used as a nanocarrier for the loading and delivery of anticancer drugs [58] such as methotrexate, doxorubicin, camptothecin [59,60], and SN−38 [61], which are commonly prescribed. In addition, loading rates of more than 200% have been reported for doxorubicin/nanoscale graphene oxide (DOX/NGO) [62]. Although the stability of GO in aqueous solution after loading with hydrophobic agents is not optimal, improvements are needed. In addition, large oxygen-containing groups at the edges or surfaces provide high chemical reactivity and modification potential for ease of preparation. Polyethylene glycol (PEG) has been widely used to functionalize rGO to increase its stability in aqueous solutions and biocompatibility [63,64]. In this study, a well-ordered AlFu–doped GO/rGO was used to efficiently deliver the saponin as a natural anticancer agent into the PDL and OSCC. Therefore, we fabricated a novel hybrid, highly porous and low-toxic saponin-loaded nanostructure by modifying graphene oxide (GO)/reduced GO (rGO) with aluminum fumarate (AlFu) as MOF core–shell nanocomposite and then loaded saponin on their surfaces. Two in vitro biological assays, including MTT assay and apoptotic evaluation, were performed to investigate the anticancer effect of these compounds on OSCC and PDL normal dental cells. The effect of the nanocomposites on OSCC was then investigated by studying apoptosis and necrosis by flow cytometry.

## 2. Results and Discussion

### 2.1. Characterization of the Developed Adsorbent

**Figure 1:**
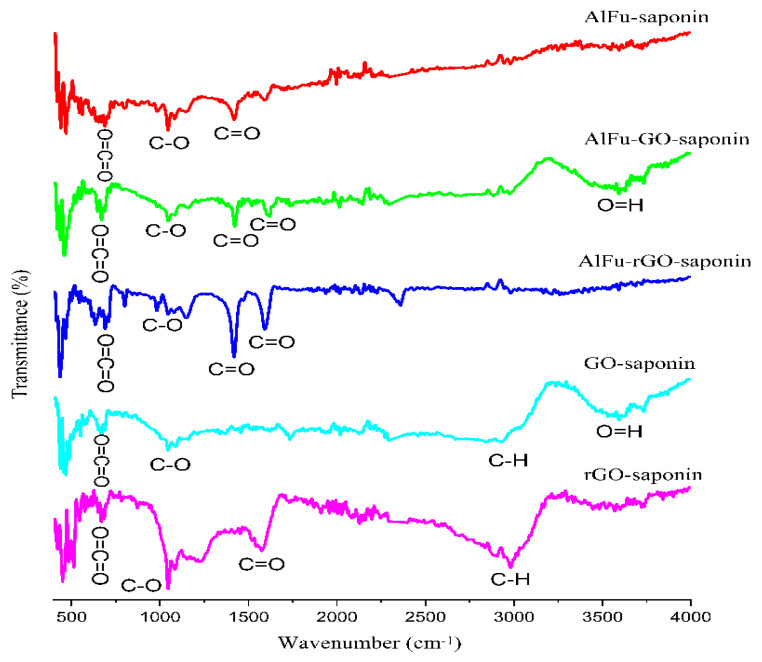
FTIR evaluation of AlFu, AlFu−GO, AlFu−rGO, GO, and rGO with saponin.

Figure 1 shows how the FTIR ranges of GO, rGO, AlFu, AlFu–GO, and AlFu–rGO can be combined. Figure 1 shows how the required graphite sources were successfully used to produce and exfoliate GO. In this case, the vibrations of a p-disubstituted phenyl bundle (v (C–H) bending in the plane), the twisting of C=C from unoxidized sp2 carbon bonds, the C=O stretching vibration (sp3 hybridization), and hydroxyl (C–OH) helpful bundles were seen in the FTIR range of 1035.70, 1577.36, 1716.29, and 3117.08 cm^−1^. In addition to the comparison with the fundamental and strongly pronounced combs, the helpful C–O (epoxy) bundles (1199.56 cm^−1^), C=C double bond carbon (1567.58 cm^−1^), and C=O stretching vibration (1731.50 cm^−1^), as well as the intense C–H band at (2963.43 cm^−1^), were also investigated in the FTIR range of rGO. [34]. The peak associated with the hydroxyl bundles disappeared, the intensity of the C=O functional group at (1724 cm^−1^) decreased, and the concentration of the C=C-related peaks increased in this region, securing and strengthening the grip for the successful reduction of graphene oxide. The results of the FTIR investigation showed that both GO and rGO nanosheets were successfully synthesized. As for AlFu, the fingerprint of Al–OH supporting bundles showed peaks in the range of 483.56–1240 cm^−1^. Other peaks in the FTIR spectrum of AlFu are due to the symmetric (Vs) or hilter-kilter (Vas) COO-extending COOs. There is also a prominent peak with a wavelength of 3385 cm^−1^, and C–O and C=O are seen at wavelengths of 1110.65 cm^−1^ and 1723.17 cm^−1^, respectively. In addition, the spectra of AlFu–GO/AlFu–rGO showed peaks at 1420.15/140.98 and 1608.19/1604.23, as well as 1120.9/1121.14 and 1721.03/1722.12 cm^−1^, which are comparable to the FTIR range of AlFu when compared with the symmetric (Vs) and asymmetric (Vas) COO-expansions of AlFu–GO and AlFu–rGO. In addition to the C–O and C=O groups seen at wavelengths of 1121.23 cm^−1^ and 1722.16 cm^−1^, hydroxyl functional groups were also found to vibrate at wavelengths of 3385.16 cm^−1^ and 3420.32 cm^−1^, respectively [33]. The combination of AlFu and its predicted composites with GO and rGO nanoflakes is supported by these data and is in perfect agreement with the results of the FTIR investigation, as can be seen in Figure 1.

**Figure 2:**
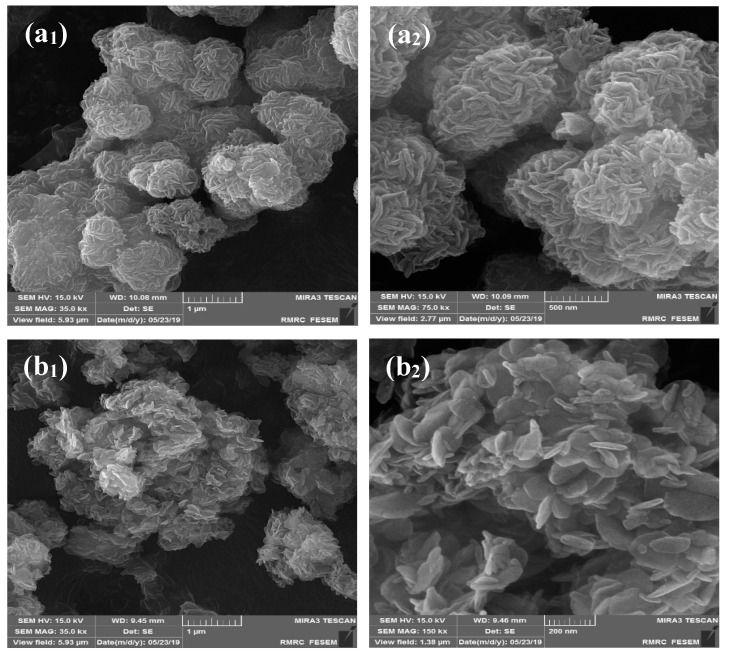

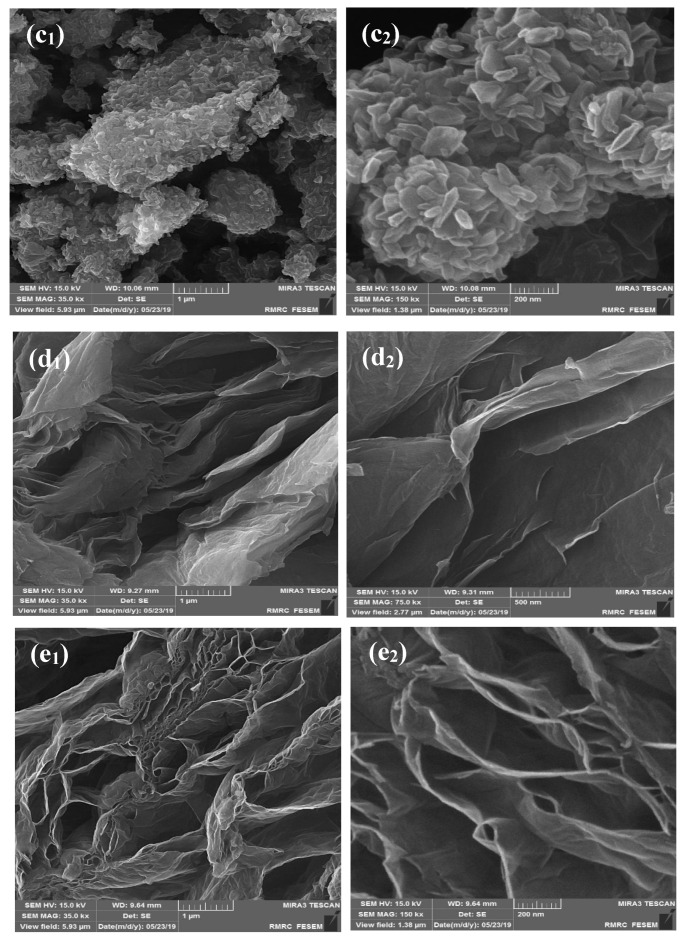

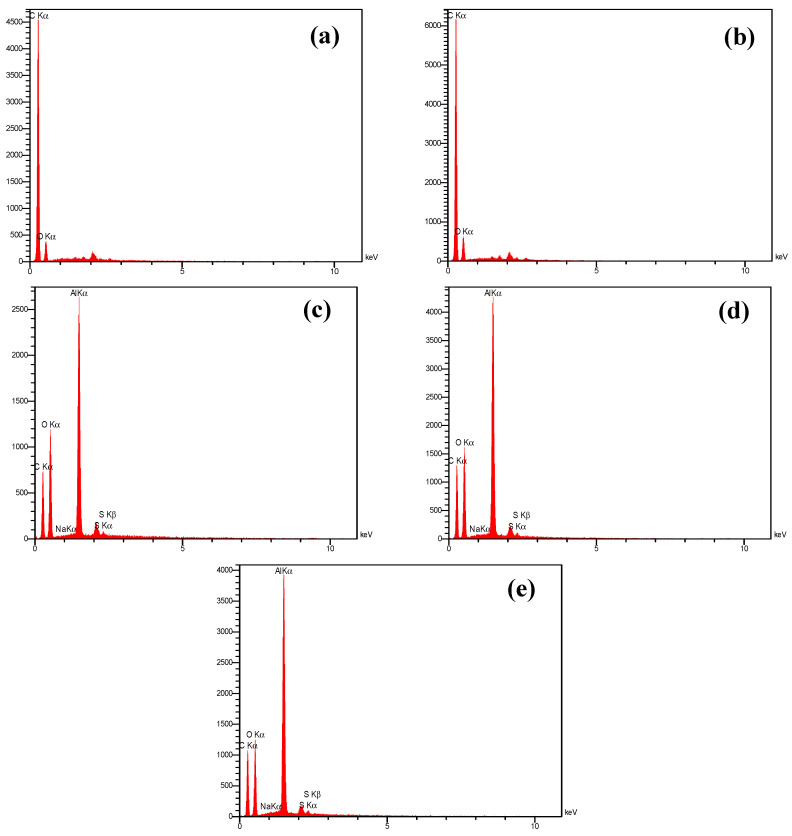
Morphology of (**a_1_**) AlFu (**a_2_**) AlFu–saponin, (**b_1_**) AlFu–GO, (**b_2_**) AlFu–GO–saponin, (**c_1_**) AlFu–rGO, (**c_2_**) AlFu–rGO–saponin, (**d_1_**) GO, (**d_2_**) GO–saponin, (**e_1_**) rGO, and (**e_2_**) rGO–saponin, through FESEM and EDX analysis of (**a**) GO, (**b**) rGO, (**c**) AlFu, (**d**) AlFu–GO, and (**e**) AlFu–rGO.

**Figure 3:**
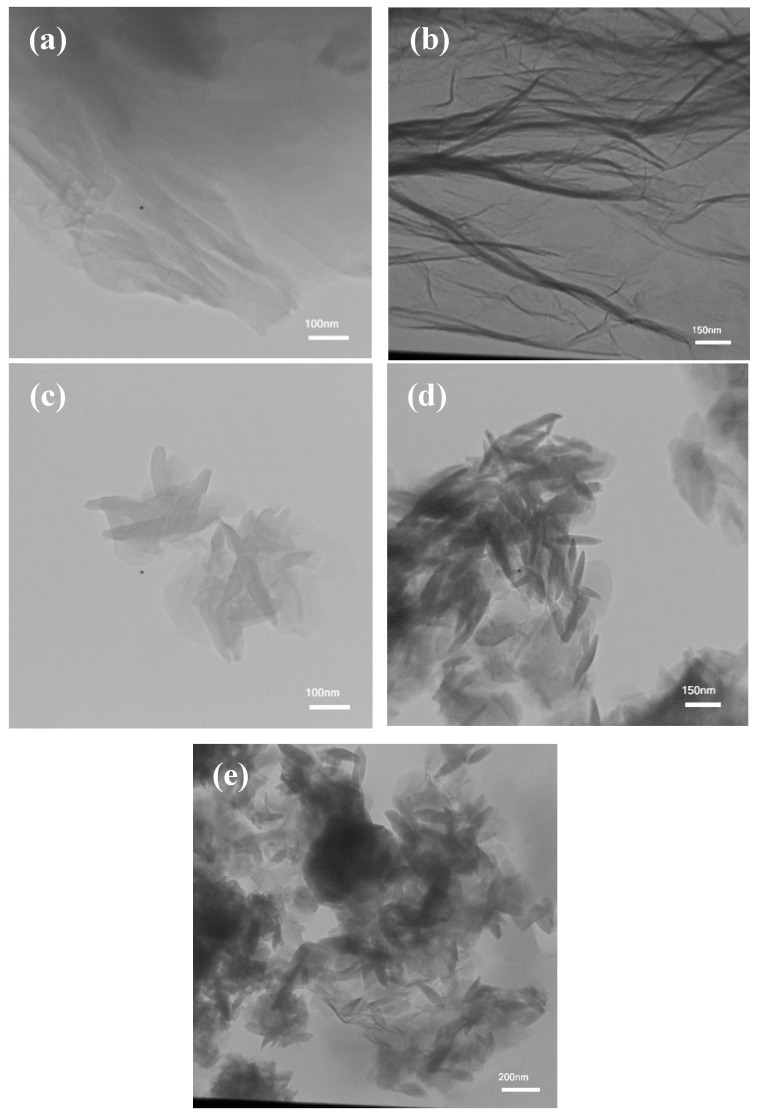
TEM images of (**a**) GO, (**b**) rGO, (**c**) AlFu, (**d**) AlFu–GO, and (**e**) AlFu–rGO.

The morphology of the nanocomposites was analyzed using FESEM. The outlines of the flawless GO, rGO, and AlFu nanocomposites without saponin are shown in Figure 2a_1_–c_1_, and the nanocomposites with saponin are shown in Figure 2a_2_–c_2_. Figure 2d_1_,d_2_,e_1_,e_2_ show a sheet-like randomly assembled slender folded layer structure for GO and rGO pure and without saponin. Figure 2a_1_,a_2_ show a layer-by-layer extended hexagonal shape in FESEM images of pure AlFu and saponin. In addition, FESEM analysis was used to investigate the sizes and shapes of the fabricated samples. Figure 2 shows this fact. As mentioned earlier, GO has a thickness range of 9–10 nm, while rGO has a thickness range of 10–16.22 nm. In addition, the FESEM images of AlFu, AlFu– GO, and AlFu–rGO pure and with saponin can be seen in Figure 2a_1_–c_2_, which show the morphology and estimated permeability of the generated nanomaterials. As can be seen from the images, octahedral AlFu nanoparticles were uniformly distributed on the surfaces of the GO and rGO flakes and enveloped the AlFu nanoparticles throughout GO. The rGO nanosheets were uniformly distributed similar to the smooth AlFu. These results, together with those from the XRD study, provide evidence for the decoration/interaction of AlFu with GO and rGO nanosheets, the exfoliation of GO and rGO nanosheets, and the arrangement of AlFu. The EDX mapping results for GO and rGO are also shown in Figure 2. Both GO and rGO appeared mainly as carbon and oxygen, while phosphorus and nitrogen were scattered randomly throughout their structures. These results demonstrate the presence of aluminum in the AlFu structure, which is crucial for the efflux and sensitivity of GO MOFs. In Figure 2a,b, AlFu and rGO appear as well-defoliated single-layer graphene droplets, which is consistent with the previously obtained data and confirms the mixture of GO and rGO. Moreover, TEM images of AlFu, AlFu–GO, and AlFu–rGO can be seen independently in Figure 3c–e. The rGO, GO, and AlFu–rGO structures show a two-dimensional wrinkled layer. This structure is formed by exfoliation and re-exfoliation processes. In this TEM study, rGO shows a thin and flexible morphology. The AlFu–GO and AlFu–rGO MOFs nanocomposite particles are shown in Figure 3d,e. The results of TEM investigation support the contact and dispersion of AlFu with and over graphene nanosheets according to the FESEM studies.

**Figure 4:**
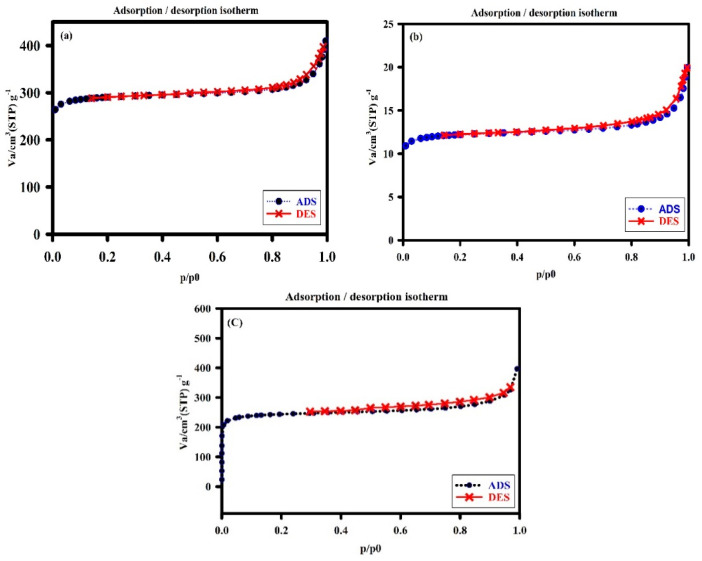
N_2_ adsorption–desorption isotherms of AlFu (**a**), AlFu−GO (**b**), and AlFu−rGO MOF (**c**) at 77 K.

**Figure 5:**
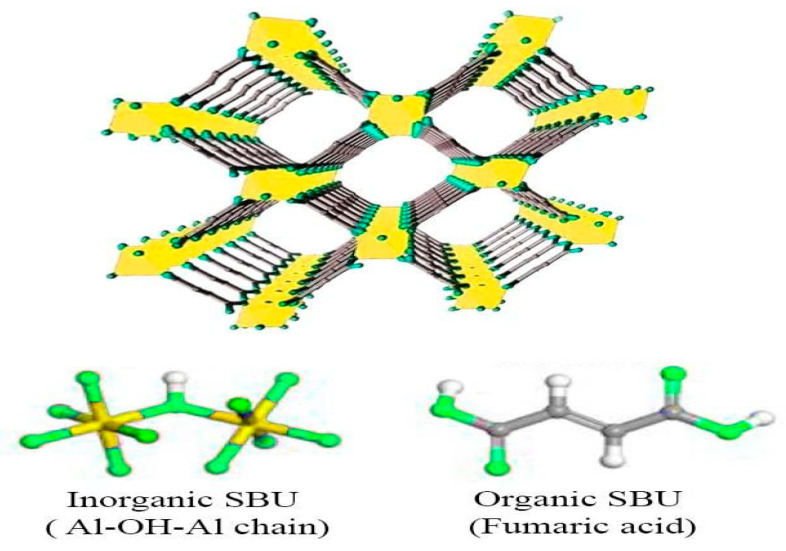
The chemical structure of the AlFu MOF.

The main specific surface area of the fabricated nanomaterials was identified by BET analysis. AlFu in this example had a single point surface area of 1011.56 m^2^/g (with a p/p° of 0.200666237), while its BET surface area, Langmuir surface area, t-plot microspore area, and t-plot outer area were 973.39, 1283.45, 869.29, and 104.09 m^2^/g, respectively. It was also found that the total surface area of pores between 17 and 3000 m^2^/g for BJH adsorption and desorption was 66.42/74.37 m^2^/g. AlFu possessed pores that were typically 22.97 nm wide and had a spacing of nearly 130.16 nm for BJH adsorption and desorption, respectively. AlFu has a single point giving a pore volume of 0.559 cm^3^/g (for pores less than 717.828 wide at a p/p° of 0.972282541), a microspore volume of 0.403996 cm^3^/g, and a BJH adsorption/desorption aggregate volume of the pores of 0.216161/0.219537 cm^3^/g, according to the primary pore volume assessment. In addition, the GO was tuned with an AlFu MOF, creating a 2D nanostructure with a suitable surface area. In this case, AlFu–GO had a pore estimate of 24.92 and had a specific single point surface area, BET surface zone, and Langmuir surface area of about 951.69, 917.79, and 1211.08 cm^3^/g, respectively. Similar to AlFu and AlFu–GO, the AlFu–rGO composite also exhibits a large surface zone, with individual surface areas, pore volumes, and raw pore diameters of 951.88 m2/g, 0.6069 cm^3^/g, and 2.5503 nm, respectively. The isotherms of nitrogen sorption and desorption of the AlFu MOF are shown in Figure 4a. The common specific surface area of the generated nanoparticles was identified by BET analysis. At a p/p° of 0.200666237, the single point surface area of AlFu was 1011.56 m^2^/g, while the area of BET surface, Langmuir surface, t-plot micropore area, and t-plot outer area were 973.39, 1283.45, 869.29, and 104.09 m^2^/g, respectively. In addition, the total surface area of the pores between 17 and 3000 m^2^/g was determined to be 66.42/74.37 m^2^/g for BJH adsorption and desorption, respectively. In terms of pore size, AlFu appears to have an average pore width of 22.97 and an average pore spacing for BJH adsorption/desorption of 130.16/118.07. According to preliminary pore volume measurements, AlFu comprises a single point that has a pore volume of 0.559 cm^3^/g (for pores less than 717.828 Å in diameter at a p/p° of 0.972282541), a micropore volume of 0.403996 cm^3^/g, and a BJH adsorption/desorption aggregate volume of 0.216161/0.219537 cm^3^/g. This fantastic information shows the dominant surface area of AlFu MOF, which makes it ideal for absorbing waste and toxins from various media. Alignment of GO with AlFu MOF also led to the formation of a 2D nanostructure with a substantially increased surface area. In this case, AlFu–GO has a single-point-specific surface area, BET surface zone, and Langmuir surface zone of nearly 951.69, 917.79, and 1211.08 m^2^/g, respectively, with a pore size of 24.92 A. The AlFu–rGO composites, such as AlFu and AlFu–GO, had high surface area with 951.88 m^2^/g, 0.6069 cm^3^/g and 2.5503 nm for the surface area, respectively, which affects the pore volume and coarse pore spacing. In this research, we investigated that AlFu MOF has a dominant surface area, which is ideal for absorbing waste and toxins from various media. As mentioned before, AlFu–GO or AlFu–rGO has a much larger surface area than AlFu, and we assume that the graphene surface area increases the total surface area of the composite, which can increase the absorption of the system. There are some methods to control the surface area of graphene that we can investigate in future research. Figure 4 shows a diagram of the AlFu MOF nitrogen sorption and desorption isotherms. As mentioned earlier, the crystal structure of AlFu MOF consists of a chain of molecular oxygen bonded to a chain of aluminum connected by fumarate linkers. The unique properties of AlFu MOF include that it has a permanently porous 3D structure of Al(OH)(O_2_C CH CH CO_2_) with square channels [56]. AlFu MOF is shown in more detail in Figure 5.

### 2.2. Biological Studies Results

**Figure 6:**
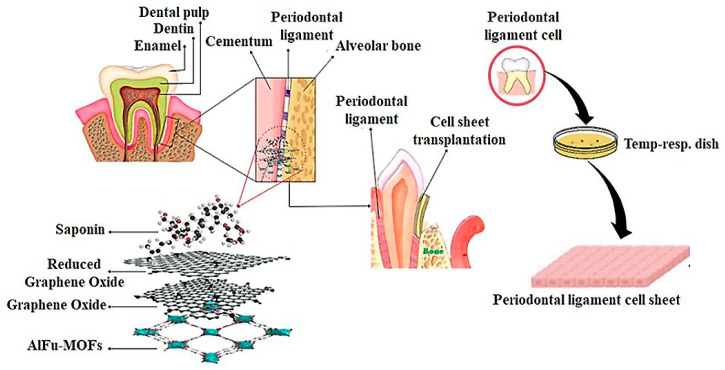
Schematic image of AlFu (MOF)/GO/rGO with saponin on PDL cell line.

**Figure 7:**
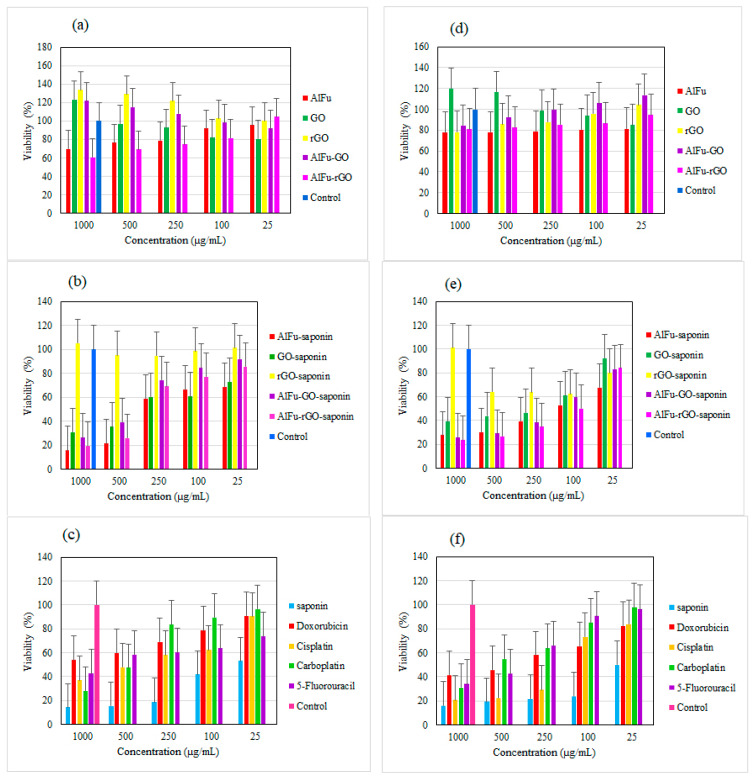
Comparison of drug toxicity and effect of AlFu (MOF)/GO/rGO with saponin on PDL cells and viability of OSCC. MTT assay results of nanoparticles (MOFs) (**a**), nanoparticles (MOFs) with saponin (**b**), and anticancer drugs (**c**) on PDL cells. MTT assay results of nanoparticles (MOFs), (**d**) nanoparticles (MOFs) with saponin (**e**), and anticancer drugs (**f**) on OSCC cells.

Recently, studies have been conducted on the anticancer and antibacterial effects of saponin [11,65,66,67]. In one study, saponin extracted from the seeds of *Madhuca longifolia* was used to clean the dental canal. The toxicity of the saponin was studied in the presence of PDL cells. The results showed that the saponin extract had a viability of 60 to 70% at concentrations of 300 µg/mL and 400 µg/mL. In contrast, sodium hypochlorite, a common substance used for dental floss, had a viability of only 22% [68]. MOFs are lattice structures of metal ions and organic ligands that exhibit good biocompatibility and complete decomposition due to their large pore surface area, adjustable pore diameter, high drug loading, stable and sustained drug release, and good biocompatibility. In vivo, they are considered as drug carriers [56]. In one study, a hybrid of reduced graphene oxide and hydroxyapatite (rGO/HA) was synthesized to produce a scaffold material for bone tissue regeneration and implantation. In addition to the hybrid substance, this study also investigated the toxicity of GO on PDL cells. The results showed that GO was not toxic, and at a concentration of 20 µg/mL, viability increased by 110% [69]. In a study of the combination of chemotherapy and photothermal therapy, an anticancer drug was loaded into GO stabilized with poloxamer 188. The results of the toxicity of GO on SCC-7 cells showed only 10% toxicity at the highest concentration of 10 µg/mL [70]. In another study, a ceramic nanocomposite of reduced graphene oxide (rGO) anchored with copper oxide was prepared by phytochemical route to investigate the antibacterial activity and anticancer properties. According to the MTT results, reduced graphene oxide (rGO) has a toxicity of less than 30% on SCC-9 cancer cells up to 150 µg/mL [71]. The synthesized nanocarriers AlFu, GO, and rGO and their hybrid compounds (AlFu–GO) and (AlFu–rGO) were investigated. The results showed low cytotoxicity and excellent biocompatibility. In addition, the low toxicity of nanocarriers to normal cells is evident in all the above studies. Figure 6 shows the schematic of AlFu (MOF)/GO/rGO with saponin on PDL cell line. The results show that the rGO sample without and with saponin has no significant toxicity on PDL cells at all concentrations. Among the investigated nanocarriers after rGO and rGO–saponin, the AlFu–GO–saponin nanocarrier showed low toxicity to PDL cells and viability of 91 ± 18.33% at a concentration of 25 μg/mL. The saponin without nanocarrier has only 53.01 ± 11.27% viability at the lowest concentration. According to the results of the MTT assay, PDL cells treated with AlFu–GO–saponin at a concentration of 250 μg/mL had a viability of 74.46 ± 16.02%, whereas OSCC cells treated with this sample at a similar concentration had a viability of only 38.35 ± 19.9%. The toxic effect of this compound on OSCC cells is clearly visible. The synthesized MOF nanocarriers with large surface area, controllable pore diameter, and high biocompatibility are very suitable for biological applications such as drug delivery (see Figure 7).

### 2.3. Comparison of the Toxicity of Nanocarriers with Saponin

The cell viability of PDL in the presence of AlFu is about 69.55 ± 15.7% at the highest concentration and about 95.53 ± 11.08% at the lowest concentration, indicating that this nanocarrier has very low toxicity to PDL cells. When saponin was combined with the AlFu–GO nanocarrier, the saponin could penetrate well into the structure of the nanocarrier, so the viability of PDL cells treated with AlFu–GO–saponin increased significantly. The viability of the AlFu–saponin sample at a concentration of 25 μg/mL was 68.66 ± 19.39%. The viability of saponin alone on PDL cells is 14.17 ± 8.92% at the highest concentration, and when saponin was combined with GO, the viability increased to 30.9 ± 17.5%. In the case of rGO, the viability of cells from PDL, similar to GO, was increased at all concentrations when it was combined with saponin, showing that the combination of this sample with the saponin has very low toxicity, in addition to the non-toxicity of rGO. The nanocarrier AlFu–GO is not as toxic as the previous three samples. Even at high concentrations, it induced the cell induction, and when the reduced concentration decreased the cell induction, the cell viability reached about 91.98 ± 21.63% when saponin was combined with AlFu–GO. When the saponin is incorporated into nanocarrier compounds, the toxicity of the saponin is significantly reduced. PDL cells treated with pure saponin at a concentration of 250 μg/mL had a viability of only 18.63 ± 8.42%, while the AlFu–GO–saponin sample at the same concentration had a viability of 74.46 ± 16.02%. In the aforementioned sample, viability increased to 91.84 ± 20.88% at a concentration of 25 μg/mL. PDL cells treated with an AlFu–rGO sample at a concentration of 1000 μg/mL had a viability of 60.62 ± 17.58%. As the concentration decreased, cell viability increased sharply and reached 104.72 ± 20.93% at a concentration of 25 μg/mL. When this sample was combined with saponin at a concentration of 25 μg/mL, a viability of 85.62 ± 18% was observed. In contrast, the viability of saponin alone at a concentration of 25 μg/mL was only about 53.01 ± 11.27%. According to the results, the AlFu–rGO nanocarrier reduced saponin toxicity at all concentrations (see Figure 7).

Figure 7 shows the percentage of viability of OSCC cells after 24 h of exposure to nanocarriers. At the highest concentrations, the toxicity of nanocarriers AlFu, GO, rGO, AlFu–GO, and AlFu–rGO is 77.6 ± 14.2%, 119.86 ± 22.64%, 78.08 ± 9.66%, 84.47 ± 13.02%, and 81.05 ± 19.44%, respectively. For the combination of AlFu carrier and saponin, cell viability of only 27.62 ± 9.11% was observed at a concentration of 1000 µg/mL. In the above sample, the viability of OSCC cells increased slightly with decreasing saponin concentration. Thus, at a concentration of 100 μg/mL, only 52.75 ± 7.48% viability was observed. The viability of the GO–saponin and rGO–saponin samples at a concentration of 500 μg/mL was 43.6 ± 12.91% and 64.15 ± 18.21%, respectively. The saponin sample alone at the same concentration has a viability of 19.17 ± 18.5%. After combining saponin with the above nanocarriers, the reduction of saponin toxicity is clearly visible. The saponin was combined with AlFu–GO nanocarrier, and the saponin was well placed in the structure of the nanocarrier, so the viability of PDL cells treated with AlFu–GO–saponin was significantly increased. According to the results of OSCC cells treated with AlFu–GO–saponin at a concentration of 1000 μg/mL, only 25.57 ± 8.04% viability was observed. As the concentration decreased, the bioavailability increased to 83.02 ± 22.29%. In addition, similar to the previous sample, the saponin compound AlFu–rGO exhibits high toxicity to cancer cells: at a concentration of 1000 µg/mL, the viability was 23.51 ± 13.12%, at a concentration of 100 µg/mL, 50 ± 13.78% of the cells were killed, and at a minimum concentration, a viability of 84.01 ± 19.96% was observed. The viability of PDL cells treated with the anticancer drugs doxorubicin-cisplatin-carboplatin-5-fluorouracil and the natural product saponin at a concentration of 1000 μg/mL was 53.86 ± 9.31%, 36.85 ± 20.1%, 28.09 ± 16.71%, 42.52 ± 14.59%, and 14.17 ± 8.92%, respectively. Viability increased with decreasing concentration, so that at a concentration of 25 μg/mL, the viability of the three drugs doxorubicin, cisplatin, and carboplatin increased to more than 90%. The highest toxicity at this concentration is associated with saponin, where the viability is only 53.01 ± 11.27%. Next, the viability of OSCC cells treated with the above anticancer drugs and saponin was examined. The results show that at a concentration of 1000 μg/mL, toxicity of about 60% was observed with all drugs. The highest degree of toxicity is associated with the saponin sample, such that half of the cells were killed at a concentration of 25 μg/mL. Due to the high toxicity of anticancer drugs to normal cells, it is necessary to use nanocarriers to reduce the side effects. The saponin used in this study is a nonionic amphiphilic saponin of natural origin. Previous studies have investigated the anticancer and antibacterial effects of the five drugs mentioned above [72,73,74,75]. In more recent studies, the effect of platycodin D, a type of triterpene saponin derived from *Platycodon grandiflorum*, on the growth and invasion of human oral cancer cells was investigated. According to the results, platycodin D at a concentration of 80 μM had no significant effect on the viability of SCC 4-SCC 9 cells after 24 h. However, 48 and 72 h after treatment, platycodin D at concentrations of 20 and 80 μM caused significant inhibition of cancer cells [76]. In another study, cold atomic plasma (CAP) was examined in vitro for reactive oxygen species (ROS) at the oral cavity cancer site induced by cisplatin, and the toxicity of cisplatin on SCC cells was also investigated. MTT results showed that cisplatin at a concentration of 30 µM had a viability of about 20% [74].

### 2.4. Flow Cytometric Detection

**Figure 8:**
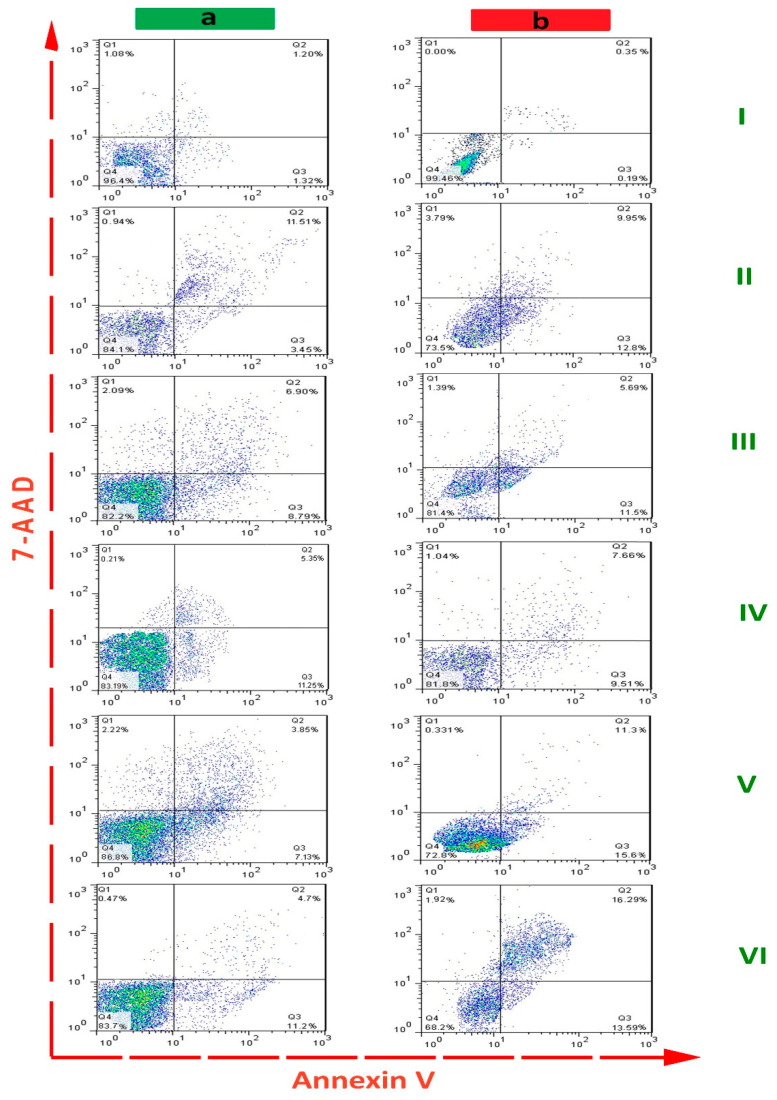
Apoptotic percent of PDL (**a**) and OSCC (**b**) cell lines after exposure to different nanostructures using flow cytometry. Annexin V+/7-AAD+ (late apoptotic), 7-AAD+ (necrotic), and Annexin V/7-AAD (live) cells in each treatment group. The groups are control (I), saponin (II), AlFu–GO (III), AlFu–rGO (IV), AlFu–GO–saponin (V), and AlFu–rGO–saponin (VI).

After treatment of PDL and OSCC cell lines with different nanostructures, the apoptotic effect was determined by flow cytometry (Figure 8). The concentration of nanostructures in each group was set at 1000 μg/mL. As shown in Table 1, the control group (untreated cells) had a minimal effect on apoptosis of PDL (2.52 ± 0.78% of total cells) and OSCC cells (1.31 ± 0.62% of total cells). In the AlFu–GO–saponin and AlFu–rGO–saponin groups, apoptotic cells were increased to 10.98 ± 2.36%–26.90 ± 3.24% and 15.9 ± 4.08%–29.88 ± 0.41%, respectively. In addition, the apoptotic cells in saponin, AlFu–GO, and AlFu–rGO groups were 14.96 ± 3.04%–22.75 ± 1.28%, 15.69 ± 3.93%–17.19 ± 2.26%, and 16.60 ± 2.54%–17.17 ± 2.80%, respectively. This significant increase in apoptotic effect observed with AlFu–rGO–saponin is also evident when compared to the nanostructures alone; thus, the nanoparticles alone have a lower effect than the combination therapy.

## 3. Materials and Method

Periodontal ligament fibroblasts (PDLs) were purchased from the Sivan (Shiraz, Iran) company. The OSCC, was purchased from (Shayan Pars Cell Bank, Shiraz, Iran). DMEM culture medium, trypsin, fetal bovine serum (FBS), phosphate buffer solution, and MTT solutions were all purchased from BIO-IDEA (Iran). The commercial saponin used in this article was “Saponin from Quillaja bark” purchased from Sigma Aldrich (Darmstadt, Germany, CAS number: 8047-15-2). The characterization of the nanostructures were investigated using different analytical techniques. The FTIR tensor II Bruker (Berlin, Germany), AlFu and its derivatives were registered in the present case. Mira 3 Tescan (Kohoutovice, Czech Republic) and Hitachi H-800 TEM (Tokyo, Japan) were used for field emission scanning electron microscopy (EDX and FESEM) to evaluate the morphology and average particle size, respectively. Surface SA-3100 was used in conjunction with a pore analyzer (Beckman Coulter, Brea, CA, USA) to determine the specific surface area of the adsorbent developed and the average pore size.

### 3.1. Preparation of GO and rGO

**Figure 9:**
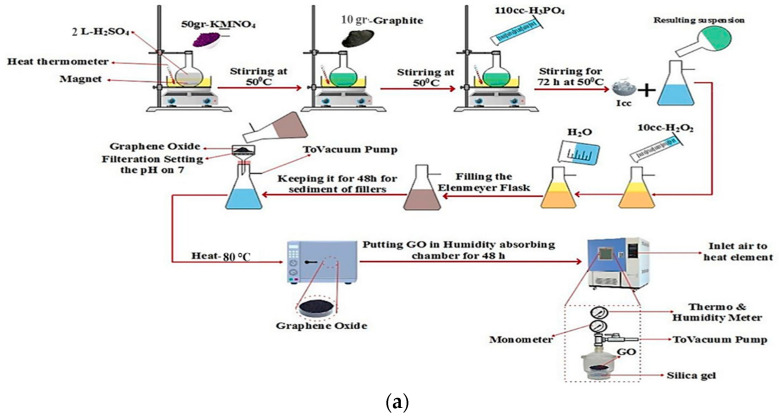

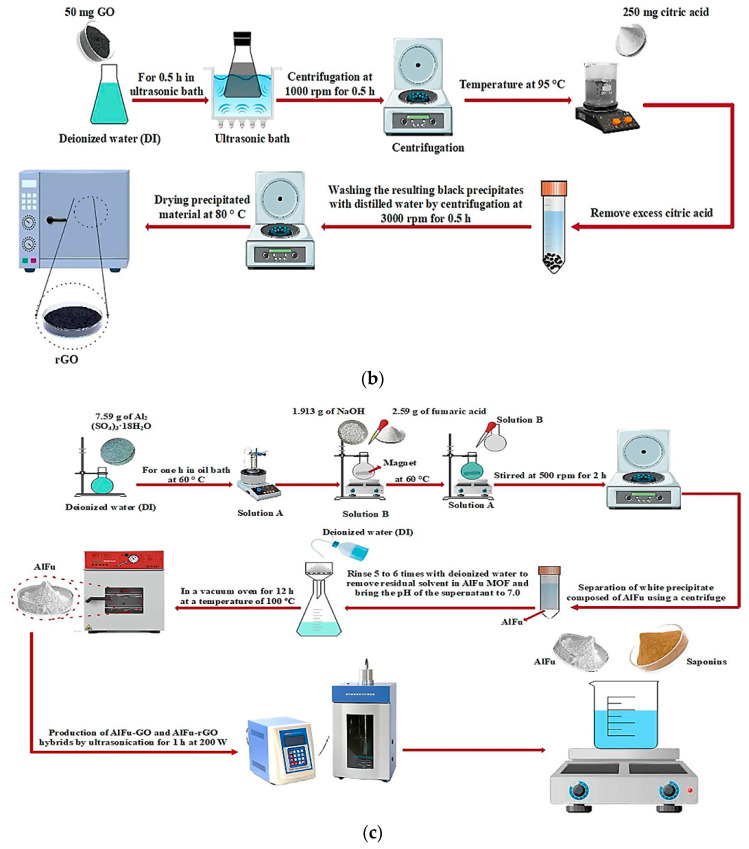
Schematic synthesis of GO: (**a**) rGO; (**b**) AlFu; (**c**) hybrid materials.

The modified Hummer’s method was used to prepare GO. First, 2 L of H_2_SO_4_ were poured into a round-bottom flask and stirred simultaneously at a temperature of 50 °C with a rotation of 300 rpm. In the next step, 50 g KMNO_4_ was added to the H_2_SO_4_ in the flask. Then, 10 g of graphite was slowly added to the stirring suspension. In the next step, 110 mL of H_3_PO_4_ was added to the above suspension and the suspension was stirred at 50 °C and 500 rpm for 72 h. Then, the suspension was transferred to an ice-cooled Erlenmeyer flask under vacuum (ice-cooled Erlenmeyer flask). In the next step, 10 mL of H_2_O_2_ was slowly added to the suspension, and the Erlenmeyer flask was filled with deionized water under vacuum. Then, the suspension was kept constant for 48 h to separate the fillers. Finally, the desired suspension was filtered, and to remove the metal ions, the remaining fillers were washed on filter paper with HCl solution. To neutralize the pH, the suspension was also washed with deionized water. In the final step, the material was dried in a heating oven at 80 °C for 1 h and then stored in a moisture-reduction chamber for 48 h. To reduce GO, the hot zone of a tube furnace is filled with fine dry powder of GO nanosheets. Ar gas is then continuously introduced into the tube while the temperature is increased by 5 °C per minute until it reaches a temperature of about 350 °C. Therefore, the GO should be kept at the above temperature for 30 min to eliminate the oxygen domains and shorten the gap. After the tube reached room temperature (RT), the exfoliated GO powder was collected and stored in the desiccator for further use. First, 50 mg of the resulting solid was sonicated in 500 mL of water in a continuous supersonic bath for 0.5 h. Because the material is highly fragmented when the tip is inserted directly into the medium, bath sonication is preferable to advanced sonication. A total of 250 mg of GO was gradually separated from the rest of the solution with vigorous shaking. Different reduction times (0.5 to 3 h) at a reduction temperature of 95 °C were investigated. To remove the excess of AC, the resulting black precipitates were washed for 0.5 h with distilled water at a rate of 3000 activities. The resulting suspension was divided into two equal parts and centrifuged at 1000 rpm for 0.5 h to produce a homogenized GO suspension (Figure 9). It was possible to use this suspension to produce rGO. Finally, rGO powder was prepared overnight and the precipitated material was dried at 80 degrees Celsius [28]. It was studied how GO dissolves. It illustrates how the primary chemical species changed over time at temperatures ranging from 1200 K to 3000 K. The degradation of GO at temperatures from 1200 K to 3000 K shows the great influence temperature has on the distribution of byproducts. At low temperatures, there are hardly any degradation products, which shows that GO is rarely decomposed. H_2_O and H_2_ are produced only in traces [77] (see Figure 9a,b).

### 3.2. Synthesis of AlFu, AlFu–GO, and rGO Hybrid Materials

AlFu MOF was prepared using the previously described technique with hydrothermal processes [50]. A round-bottom flask containing 32 mL DI and 7.59 g Al_2_ (SO_4_)_3_–18H_2_O was heated at 60 °C for one hour over some exotherm (labeled solution A). Then, 1.913 g NaOH was suspended in 38.5 mL deionized water (DI) and 2.59 g fumaric acid (C_4_H_4_O_4_) was added dropwise. Stirring was then performed until a homogeneous suspension (designated solution B) was formed. To precipitate the white AlFu precipitate, solution B was gradually added dropwise to solution A at 60 °C. The combination was then stirred at 500 rpm for two hours. To remove any solvent still in the AlFu MOF and to bring the pH of the supernatant solution to 7.0, the unreacted reagents were washed five to six times with deionized water. The materials were then dried at 100 °C for 12 h in a vacuum oven. AlFu–GO and AlFu–rGO hybrids were prepared by dispersing a certain amount of GO and rGO powder in the initial water of solution A (32 mL DI) at 200 W for one hour. The same AlFu procedure was then used to synthesize AlFu–GO and AlFu–rGO in the presence of GO and rGO suspensions, and the efficacy of the prepared AlFu, AlFu–GO, and AlFu–rGO against OSCC was investigated. The four anticancer drugs used in the control groups were cisplatin, carboplatin, doxorubicin, and 5-fluorouracil (see Figure 9c).

### 3.3. Toxicity Evaluation

In this study, OSCC and PDL cell lines were used to evaluate cytotoxicity in vitro. Experiments with the cells were performed in triplicate. In this study, DMEM was used as cell culture medium supplemented with 10% fetal bovine serum (FBS) and 5% penicillin–streptomycin. Cells were cultured at 37 °C in a humidified environment with 5% CO_2_ gas before experimental evaluation. Subsequently, a 3-(4,5-dimethylthiazol-2yl)-2,5-diphenyltetrazolium bromide (MTT) assay [77] was performed to evaluate the cytotoxicity of the AlFu–GO hybrid composite against OSCC and PDL. For this purpose, the cultured cells were seeded on 96-well plates with approximately 2000 cells per well. In the first phase, OSCC and PDL were incubated for 24 h, followed by treatment of the cells with AlFu–GO and AlFu–rGO by saponins at a concentration range of 25, 100, 250, 500, and 1000 µg/mL. Cells were then incubated at 37 °C and 5% CO_2_ for 24 h. A 5 mg/mL MTT stock solution was then prepared using PBS. This stock solution was then diluted with DMEM medium without phenol to a concentration of 0.5 mg/mL (1 mL MTT stock solution (5 mg/mL) + 9 mL phenol-free DMEM). After aspirating the old medium from each well, 30 μL of the diluted DMEM–MTT solution was added to each well. The plates were incubated at 37 °C and 5% CO_2_ for 3 h. Then, 100 μL of DMSO was added to the wells to dissolve the insoluble formazan crystals. Finally, the microplate reader (BioTEK Power Wave XS2) was used to determine the absorbance of the respective solutions at the main wavelengths of 570 nm and the reference wavelengths of 630 nm. In this paper, the reference wavelength was subtracted from the original wavelength (A570–A630) in the calculations. Therefore, the reference wavelength was used to exclude some factors such as precipitated proteins or cell debris in the wells.

$$Viability\;\left(\%\right)=\frac{A_{t}-A_{b}}{A_{c}-A_{b}}\times 100$$

where *A_t_* corresponds to adsorption in each test well, *A_b_* corresponds to adsorption in the blank well (this well includes media + MTT + DMSO; no cells are cultured in this well), and *A_c_* corresponds to adsorption in the control well.

### 3.4. Flow Cytometric Detection of Apoptosis/Necrosis

The extent of apoptosis in OSCC and PDL cells after treatment was determined by staining with APC-annexin V and 7-AAD (BioLegend, London, UK), because the nanoparticles were shown not to interact with these channels. The medium and detached cells were stained with 50 µL APC-Annexin V (2.5 µL/mL annexin binding buffer) for 30 min after being exposed to the tested compounds for 24 h. Cells were labeled and then washed three times with annexin-binding buffer before being placed again in a staining solution containing 2.5 µL/mL 7-AAD. The FACSCanto II flow cytometer from BD Biosciences, San Jose, California, USA, was then used to analyze the cells (10,000 events per treatment). FlowJo 10 and GraphPad Prism 7 software programs were used to analyze the data (gating strategy). The cells treated with 10 µL of DMSO served as a control group in this assay (e.g., untreated group).

### 3.5. Statistical Analysis

The biological studies, e.g., MTT assay and apoptotic assay, were performed in triplicate for each study group. Data were compared by one-way analysis of variance (ANOVA) followed by Tukey post hoc test (SPSS software, IBM version 23, IBM Corp., Armonk, N.Y., USA). The significance level was set at a *p*-value ≤ 0.05.

## 4. Conclusions

We explored the concept in this study using the development of AlFu MOF and the resulting crossover nanocomposites with GO and rGO nanosheets. In this case, the fabricated specimens were accurately characterized, showing that they were successfully synthesized and exhibited a fine crystalline structure. According to the results of the conducted studies, AlFu, AlFu–GO, and AlFu–rGO exhibit a predominant porosity in the high beta surface area of about 973.39, 917.79, and 951.88 m^2^/g, respectively. This property makes them ideal candidates for highly efficient and precise biotherapeutic applications. Saponin and AlFu can work better than routine and basic drugs, such as doxorubicin, cisplatin, carboplatin, and 5-fluorouracil, in areas of PDL cells, which significantly reduces their side effects and improves their anticancer performance. In conclusion, this study proposes an orderly protocol for the precise development and production of anticancer agents for OSCC cancer, which should be performed prior to in vivo and in vitro evaluations to achieve maximum performance with the fewest side effects. In addition, evaluation of efficacy and safety of new anticancer drugs is critical and should be studied before the drug is forwarded for further analysis.

## Figures and Tables

**Table 1:** Comparison of early apoptotic, late apoptotic, and cumulative apoptotic cell populations of PDL and OSCC cell lines in each treatment group.

Compound	Early Apoptosis (%)		Late Apoptosis (%)		Cumulative Apoptosis (%)	
	PDL cell line	OSCC cell line	PDL cell line	OSCC cell line	PDL cell line	OSCC cell line
control	1.20 ± 0.45	0.35 ± 0.81	1.32 ± 0.08	0195 ± 0.31	2.52 ± 0.78	1.31 ± 0.62
saponin	11.51 ± 0.14	9.95 ± 1.05	3.45 ± 0.41	12.80 ± 2.57	14.96 ± 3.04	22.75 ± 1.28
AlFu–GO	6.9 ± 3.39	5.69 ± 1.85	8.79 ± 0.74	11.5 ± 1.33	15.69 ± 3.93	17.19 ± 2.26
AlFu–rGO	5.35 ± 0.58	7.66 ± 0.96	11.25 ± 3.12	9.51 ± 0.51	16.60 ± 2.54	17.17 ± 2.80
AlFu–GO–saponin	3.85 ± 0.07	11.30 ± 0.07	7.13 ± 1.57	15.60 ± 0.16	10.98 ± 2.36	26.90 ± 3.24
AlFu–rGO–saponin	4.7 ± 1.90	16.29 ± 3.48	11.2 ± 1.43	13.59 ± 2.11	15.9 ± 4.08	29.88 ± 0.41

## Data Availability

Data is contained within the article.
